# Floodplain soils contamination assessment using the sequential extraction method of heavy metals from past mining activities

**DOI:** 10.1038/s41598-022-06929-7

**Published:** 2022-02-21

**Authors:** Radoslava Kanianska, Jozef Varga, Nikola Benková, Miriam Kizeková, Ľubica Jančová

**Affiliations:** 1 Faculty of Natural Sciences, Matej Bel University Banská Bystrica, Tajovského 40, 974 01 Banská Bystrica, Slovakia; 2 National Agricultural and Food Centre, Grassland and Mountain Agriculture Research Institute, Mládežnícka 36, 974 21 Banská Bystrica, Slovakia

**Keywords:** Biogeochemistry, Environmental sciences

## Abstract

Floodplains are among the most precious and threatened ecosystems in the world. The study deals with floodplain soil contamination caused by 8 heavy metals (HMs) (Cd, Co, Cr, Cu, Mo, Ni, Pb, Zn) originating and transported from old mine works along the Štiavnica River in Slovakia. We determined the total HMs content and the HM fractions using BCR sequential extraction method. We selected 12 alluvial sites (AS), two contaminated sites (CS), and one reference site (RS). The sampling points were located within the riparian zones (RZ), arable lands (AL), and grasslands (GL). We confirmed soil contamination by HMs and the related ecological risk by different factors. The contamination by HMs at many AS localities was similar or even higher than at CS localities. The highest contamination factor was calculated for Cu (39.8), followed by Pb (27.4), Zn (18.2), and Cd (7.2). The HMs partitioning in the different fractions at the CS and AS localities revealed that Cd, Zn, and Pb were mainly associated with the exchangeable and reducible fractions, while Cu was mainly associated with the oxidisable fraction. The soil properties were selectively correlated with the HM fractions. Based on the ANOVA results, the effect of different ecosystem types on HM fractions was revealed.

## Introduction

Floodplains are relatively flat land surfaces created when alluvial material carried by surface water is deposited. Rivers and floodplains are connected, exchanging water, sediments, biota, and nutrients in a shared natural ecosystem. In the natural state, floodplains are characterised by high biodiversity with the presence of riparian areas, natural or artificial grasslands, and agricultural land. Floodplains fulfil many ecosystem services, including regulating such as filtration. They cover 7% of the total European area and host 12% of the European population. In Slovakia, more than 25% of the population lives in floodplain areas^1^. Floodplains are among the most dynamic, productive, diverse, and precious ecosystems in the world^2,3^. On the other hand, floodplains are the most threatened ecosystems in the world. In degraded floodplains, the quality of ecosystem services is reduced. In Europe and North America, up to 90% of floodplains are cultivated and, therefore, functionally extinct. In countries with fast growing economies, there is a huge discharge of pollutants into floodplains that are so vulnerable and highly threatened^4^.

Today, many river floodplains are highly contaminated by HMs that exceed standards for soils and sediments and also the filtering capacity of alluvial soils. Pollution is a very prominent and lasting environmental problem around the world, despite the efforts made in recent decades to clean up the environment. Soil contamination in Europe is a great challenge and puts human and environmental health at risk. Soil contamination is mainly located near waste landfills and industrial activities that spread heavy metals (HMs) and other potentially toxic elements. Important anthropogenic sources of HMs are present in former mining activities, foundries, smelters, and other diffuse sources^5^. Historically, the mining industry is one of the oldest documented types of human activity. Mining waste rocks and mining tailings contain chemicals and residues characterised by a high content of HMs. Mining activities as the main source of HMs constitute a real concern around the world, and many researchers study their impact on soil pollution^6–9^. HMs contained in mining waste can be released from the mining field into the environment and can be dispersed by gravitational transport and fluvial pathways. Floods are directly involved as serious agents of contaminant dispersion^10,11^, resulting in sedimentation on lands, where contaminants can remain for decades and centuries until they are remobilised by surface or river bank erosion^12^.

In floodplain ecosystems, soils provide important filtration services. Soils remove different contaminants, including HMs from water and prevent their bioavailability to plants and animals. Nevertheless, floodplain soils still have received little attention and their potential contamination is often underestimated or ignored. Little attention is also devoted to a deeper analysis of ecosystem services based on analytical work. The reason is that traditional floodplain management has emphasized the conversion of floodplains to uses such as agriculture, housing, and industry^13^. This emphasis on management has also had enormous economic benefits through the development of intensive agriculture on rich alluvial soils neglecting the real environmental problems and the need to protect soils and their services.

Contaminated floodplain soils and sediments pose a potential danger to the safety of agricultural products and human health, and can adversely affect the environment and ecosystems^14^. There are different studies around the world that focus on contaminated floodplains^15–17^. The large variation in contaminants, soil properties, and other natural and land use conditions at floodplains makes it impossible to assess the full extent of soil pollution and related filtration ecosystem services (FES). In addition, different contaminants have different effects on ecosystems, living organisms, human health, and the environment depending on their properties. The effect of the contaminant depends not only on its quantity, but also on its dispersion potential, solubility, and bioavailability. The total HMs content of soils is useful for many geochemical applications, but the fractions of these metals are often of greater interest. Different fractions exert different environmental effects, directly affecting the toxicity of metals, their migration, and natural cycling^18^. We suppose that the determination of different HM fractions in the soil together with the analysis of the relations between HM fractions and soil properties is possible to assess the FESs and their related capacity.

Therefore, regional studies can be a valuable contribution to a complex mosaic of knowledge about soil pollution and soil FESs. Understanding the identification of heavy metal forms and their interactions with the physical, chemical, and biological properties of soil is the necessary knowledge for a more proper and sustainable ecosystem-based management of floodplains.

The aim of this study is to investigate the contamination of the Štiavnica River floodplain by HMs originated from past mining activities, and: (1) to quantify the total content and the various fractions of HMs in soils with an emphasis on mobile and bioavailable fractions; (2) to assess the environmental impact of HMs and the degree of soil pollution with various factors; (3) to determine the relationships between different soil properties and fractions of HMs that affect the FES capacity of the floodplain ecosystem.

## Material and methods

### Sampling sites and soil sampling

Sampling sites were selected in the floodplain along the Štiavnica River in central Slovakia. The riverbanks consist of fluvial deposits (sand and silt). The Štiavnica River is 55 km long, with a 443 km^2^ large catchment area. In the upstream part of the basin in the Štiavnické vrchy Mounatins of volcanogenic origin, the relief is dominated by hills and valleys with forests and partly by permanent grasslands used as meadows. The bedrock consists mainly of andesites and rhyolites with scattered occurrences of conglomerates and shales. In the downstream part of the basin in the Podunajská pahorkatina Hills, the relief is dominated by a flat surface. The bedrock consists of clay- and sand-stones on slopes, sand and gravel in areas of floodplains. The land is used as arable land. Due to volcanogenic origin, the region of the Štiavnické vrchy Mountains is mineral-rich. The extraction of precious metals in the central part of the Štiavnické vrchy Mountains has a very long history. Large-scale mining started in the twelfth century. The discovery of precious metals encouraged rapid expansion. Silver ore prevailed among the mined metals. In the eighteenth century, the region became the biggest mining centre in the Habsburg Monarchy. In the period between 1790 and 1863, the mountains yielded 490 metric tons of silver and 11 tons of gold. At that time, there was no environmental legislation preventing the release of contaminated water and sediments into watercourses. At the end of the nineteenth century, mining fell in decay. This trend also continued in the twentieth century. Past mining activities left imprint on the environment, including contamination by HMs and other potentially toxic elements. Nowadays, the storage and handling of tailings is a major environmental issue in the region. There exist several old mine tailings, waste heaps, and dumps. The ore field is drained by the Štiavnica River.

**Figure 1:**
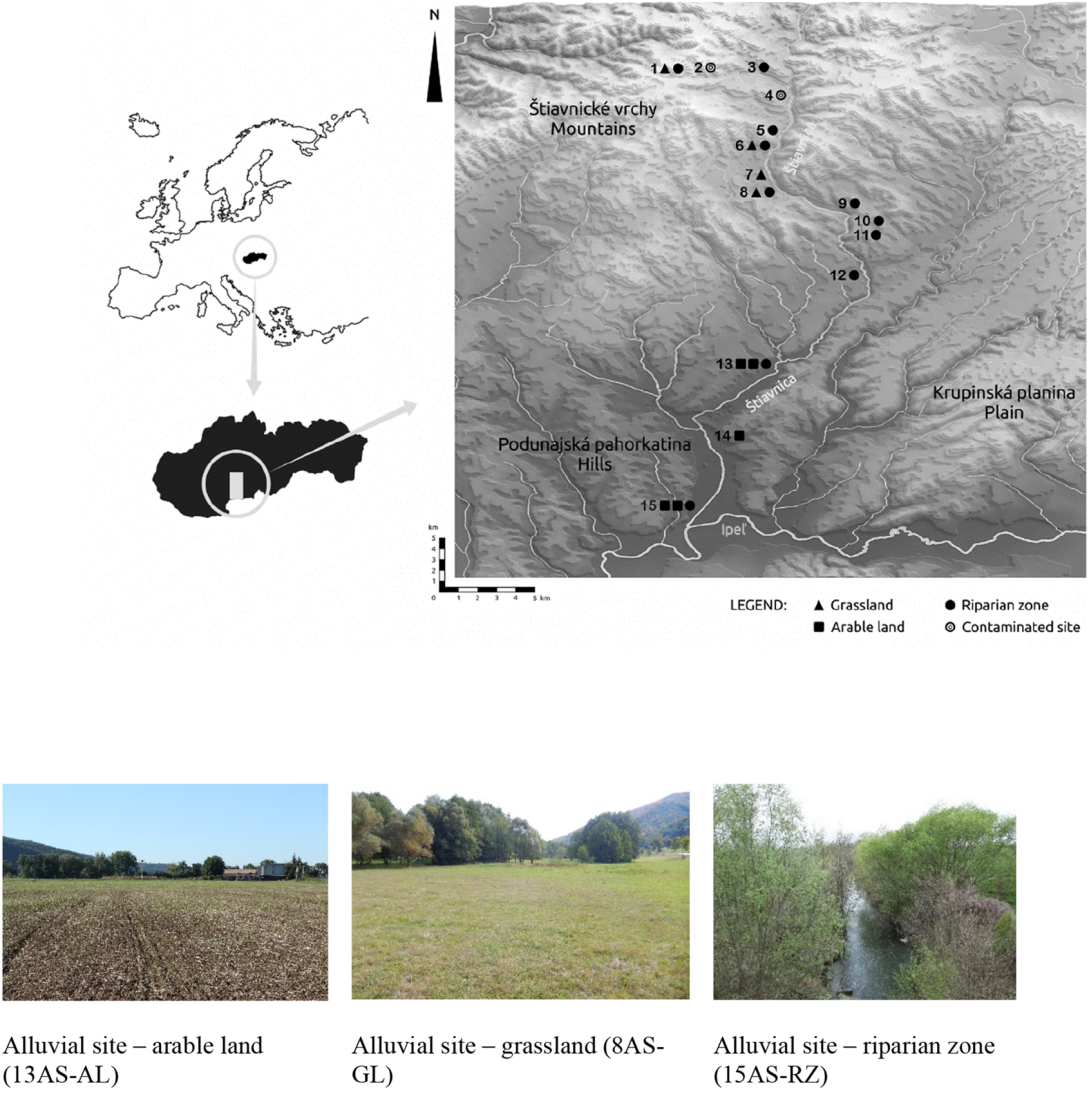
Isometric map of the sampling points and photos from selected study sites (photos R. Kanianska). The map was generated using 3D Map Generator, ver. 1.5, https://www.3d-map-generator.com/3d-map-generator-terrain/.

To study the contamination of floodplain soils by HMs from past mining activities and transported by the Štiavnica River and its sediments, we selected a total of 15 study sites along the Štiavnica River, including 2 contaminated sites (2CS, 4CS), 1 reference site (7RS) and 8 alluvial sites (AS) located in the Štiavnické vrchy Mountains (1AS, 3AS, 5AS, 6AS, 8–11 AS), and 4 alluvial sites situated in the Podunajská pahorkatina Hills (12–15 AS) (Fig. 1).

The soil types on the floodplain were classified as Haplic Fluvisols according to the World Reference Base for Soil Resources. The sampling points were located on the river bank within the riparian zones (RZ) in the Štiavnické vrchy Mountains, and on arable land (AL) in the Podunajská pahorkatina Hills. In the case of a larger floodplain, we also collected samples distant from the water flow at the end of the floodplain, that were used as permanent grasslands (GL) in the Štiavnické vrchy Mountains, and as arable land in the Podunajská pahorkatina Hills (at 5 of 12 alluvial sites). Outside the river floodplain, we selected the two contaminated sites (CS) classified as Ekranic Technosols, which are poorly developed on metallurgical slag and shallow soils. As CS sites, we selected the area of the Maximilian shaft that is registered in the Register of environmental burdens of the Slovak republic as probable environmental burden (PEB), and the hard rock mine tailing Lintich that is registered as a confirmed environmental burden (CEB), where hazardous substances caused by mining activities can pose a risk to human health or to the environment, soil and groundwater. The Maximilian shaft area was located approximately 1 km and the Lintich mine tailing approximately 100 m from the Štiavnica River. The RS site represents a site with a ´normal´ level of contaminants, and arising from a combination of natural and diffuse pollution contributions. The RS site was used to determine the background values. The RS site was located about 400 m away from the Štiavnica River outside the floodplain on a slope, and is used as a meadow. The soil type was classified as Haplic Cambisol.

### Soil analysis

Soil samples were collected in September 2018 from a depth of 0–10 cm, and 20–30 cm (total of 123), according to the soil profile (presence of bedrock). For the total content of 8 HMs (Cd, Co, Cr, Cu, Mo, Ni, Pb, Zn) in the soil was used as the digestion medium the mixture of HNO_3_/HF/HClO_4_. Then, we applied a three-step sequential extraction scheme introduced by the European Commission through the BCR Programme called the Community Bureau of Reference (BCR) as described by Rauret et al.^19^. The three-steps sequential extraction method operationally defines metals in four chemical forms: (I) Exchangeable and weak acid soluble fraction (HOAc extractable fraction), (II) Reducible fraction (bound to Fe and Mn oxides, extraction with NH_2_OH-HCl), (III) Oxidisable fraction (bound to organic matter and sulphides, digestion with H_2_O_2_ was followed by extraction with NH_4_OAc), and (IV) Residual fraction (bound to silicate minerals). The residual fraction was calculated as the difference between the total content of HM and the sum of the three metal fractions. The procedure followed was inductive coupled plasma optical emission spectrometry (ICP-OES). To guarantee data quality, reagent blanks were used. We measured the Eh in the soil using the Oxygen Diffusion meter (Ejkelkamp). We further determined soil chemical properties such as pH in H_2_O (with a ratio of 1:2.5) and 1 M KCl solution (with a ratio of 1:2.5), total soil organic carbon (SOC) using the oxidimetric method according to Tyurin that is similar to the Walkley–Black oxidation method. The Tyurin titrimetric method is a wet combustion method, where soil organic matter is oxidized by 0.2 M K_2_Cr_2_O_7_ with H_2_SO_4_. After oxidation, excess dichromate is determined by titration with a Mohr´s salt solution. The content of humic and fulvic acids was determined by the short fractionation method according to Kononova and Belchikova using extraction with 0.1 M Na_4_P_2_O_7_·10H_2_O. Oxalate-extractable Al, Fe and Mn as an indicator of hydrous oxides were determined by the acid oxalate extraction method described by Pansu and Gautheyrou^20^ with a (NH_4_)_2_-oxalate solution adjusted to pH 3, shake for 4 h in the dark and centrifuge for 10 min at 10,000×*g*.

The hydrological soil properties were determined using overall undisturbed soil samples using core-extracting tubes (Eijkelkamp Equipment for Soil Research, The Netherlands). Undisturbed soil samples in tubes were taken to the laboratory for hydrophysical analyses by the gravimetric method of particle density (PD), bulk density (BD), maximum capillary water capacity (MCWC), field water capacity (FWC), actual soil moisture (SM), porosity (as total—Pt, capillary—Pc, non-capillary—Pn, semi-capillary—Ps), and minimal air capacity (MAC) according to Novak. Particle size analysis was performed by pipette method using soil particle sedimentation and particle size fractions (sand, silt, clay) were classified according to the United States Department of Agriculture (USDA) system.

### Data analysis

The degree of the soil pollution and the related ecological risk we described with the help of contamination factor (CF), pollution load index (PLI), potential ecological risk of individual metals (Er), and potential ecological risk of the environment (RI).

The CF also designated as an enrichment factor was calculated according to the formula:

$$\mathrm{CF}=\frac{\mathrm{Ci }}{\mathrm{Cb}}$$

where Ci is the measured content of HM in soil, and Cb is the background value of the HM contents of the reference site. According to Varol^21^, the CF < 1 means low contamination, 1–3 is moderate contamination, 3–6 is considerable contamination, and CF > 6 means very high contamination.

To identify heavy metal contamination as a total scale in soil, Tomlinson et al.^22^ recommended an equation for calculating the pollution load index (PLI):

$$\mathrm{PLI}=\sqrt[\mathrm{n}]{\mathrm{CF}1*\mathrm{CF}2*\mathrm{CF}3\dots \mathrm{Cfn}}$$

where n is the number of metals. PLI > 1 indicates pollution and demonstrates dynamic deterioration of quality.

According to Hakanson’s^23^ methodology, the potential ecological risk (Er) of a given heavy metal is defined as follows:

$${\mathrm{Er}}^{\mathrm{i}}={\mathrm{Tr}}^{\mathrm{i}}*{\mathrm{CF}}^{\mathrm{i}}$$

where Tr is the toxic response factor for a given substance, CF is the contamination factor. The Tr was suggested by Hakanson^23^ for six metals Cd (30), Pb (5), Hg (40), Cu (5), Ni (5), Zn (1). The sum of individual potential risks (Er) is the potential ecological risk of the environment (RI) of the study site:

$$\mathrm{RI}=\sum {\mathrm{Er}}^{\mathrm{i}}$$

The Er < 40 means low risk, 40–80 is moderate risk, 80–160 is considerable risk, 160–320 is high risk, and > 320 is very high risk. The RI < 150 means low risk, 150–300 is moderate risk, 300–600 is considerable risk, and > 600 is very high risk.

To identify the relationships between HMs content and soil parameters, statistical analyzes were conducted using the Pearson correlation coefficient. Hierarchical cluster analysis (HCA) was applied to group similar localities (spatial variability) using the Ward´s method and Euclidean distances. A two-way ANOVA analysis was performed to assess statistically significant differences in HM fractions between different types of ecosystems (riparian zone, alluvial grassland, arable land), and soil depths (0–10, 20–30 cm). The SPSS Statistics 19 software was used to perform statistical analysis of the data.

## Results

### Basic characteristics of soils

The basic statistical characteristics of the chemical and physical properties of the soils were tested in the laboratory and calculated at the CS, AS, and RS sites in two depths. Data are reported in Tables 1, 2. There are differences between the observed properties at specific sites (CS, AS, RS) and in different depths, but also between AS sites themselves, confirming their heterogeneity.

**Table 1:** Soil chemical properties in the soil depth of 0–10 and 20–30 cm in contaminated, alluvial and reference sites with basic statistical characteristics.

Depth (cm)	Site		pH/H_2_O	pH/KCl	Eh (mV)	SOC (g/kg)	HA/FA	Al_o_ (mg/kg)	Fe_o_ (mg/kg)	Mn_o_ (mg/kg)
0–10	CS (n = 2)	x̄	7.5	7.2	n.a.	24.30	0.57	1373.8	11,398.5	4817.9
		SD	0.1	0.2	n.a.	9.30	0.02	573.4	4312.0	3708.5
		Min	7.4	7.0	n.a.	15.00	0.55	800.4	7086.6	1109.4
		Max	7.6	7.4	n.a.	33.60	0.58	1947.2	15,710.5	8526.3
	AS (n = 19)	x̄	6.3	5.5	351.95	23.08	0.43	1327.0	8280.2	2838.4
		SD	0.6	0.8	151.80	6.84	0.07	403.9	1338.7	2774.8
		Min	5.4	4.4	− 274.00	11.25	0.31	931.7	6255.8	622.0
		Max	7.7	7.2	484.00	36.60	0.59	2164.2	10,933.5	10,829.5
	RS	x	5.7	4.6	364.00	34.95	0.32	2625.7	2876.4	740.5
20–30	AS (n = 12)	x̄	6.3	5.4	n.a.	16.95	0.44	1478.	10,364.3	3285.5
		SD	0.8	1.0	n.a.	6.02	0.11	436.4	2733.4	3758.3
		Min	5.0	4.1	n.a.	6.75	0.32	941.8	6855.4	628.8
		Max	7.6	7.1	n.a.	32.25	0.68	2496.5	15,455.4	13,448.7
	RS	x	5.8	4.7	n.a.	22.50	0.37	2914.4	3518.0	991.9

*AS* alluvial site, *CS* contaminated site, *RS* reference site, _*o*_ extracted by oxalate.

**Table 2:** Soil physical properties in the soil depth of 0–10 and 20–30 cm in contaminated, alluvial and reference sites with basic statistical characteristics.

Depth (cm)	Site		Content of size particles %			PD (g/cm^3^)	BD (g/cm^3^)	MCWC (%)	FWC (%)	SM (%)	Pt (%)	Pc (%)	Pn (%)	Ps (%)	MAC (%)
			Clay	Silt	Sand										
0–10	CS (n = 2)	x̄	7.59	36.95	55.46	2.33	1.26	23.75	20.35	19.86	45.83	20.35	20.40	5.08	22.08
		SD	0.30	5.14	5.44	0.10	0.08	0.57	0.74	0.54	1.06	0.74	1.41	0.39	1.63
		Min	7.29	31.81	50.02	2.23	1.18	23.17	19.60	19.32	44.77	19.60	18.99	4.69	20.45
		Max	7.89	42.09	60.90	2.43	1.34	24.32	21.09	20.40	46.89	21.09	21.82	5.47	23.72
	AS (n = 19)	x̄	10.22	47.54	42.18	2.25	1.10	36.99	32.37	30.17	51.36	32.37	13.56	5.43	14.37
		SD	8.05	16.58	20.63	0.14	0.21	8.83	8.68	12.12	8.48	8.68	13.19	5.29	12.36
		Min	4.45	19.05	4.32	1.91	0.72	22.30	20.26	15.68	35.59	35.59	20.26	0.00	0.00
		Max	42.28	77.76	76.62	2.59	1.50	57.20	51.65	57.09	66.16	66.16	51.65	39.37	46.66
	RS	x	25.81	51.48	22.71	2.43	1.34	34.70	32.28	19.96	36.29	32.28	0.17	3.84	1.59
20–30	AS (n = 12)	x̄	10.63	40.00	49.38	2.26	1.19	36.95	31.98	29.03	46.82	31.98	8.51	*6.33*	9.87
		SD	*9.12*	15.63	21.16	0.19	0.28	8.96	11.07	13.45	13.40	11.07	10.31	5.09	10.31
		Min	4.35	22.78	8.53	2.04	0.69	30.35	18.67	10.88	30.95	18.67	0.00	0.23	0.00
		Max	39.43	78.81	72.87	2.67	1.54	58.37	56.44	58.12	67.47	56.44	24.72	17.28	27.14

*AS* alluvial site, *CS* contaminated site, *RS* reference site.

### Soil contamination by heavy metals

The total HM content in the two soil depths of the Štiavnica River floodplain together with the Slovak, Finish and Canadian guideline values of the contaminated soil are presented in Table 3. The Finish legislation sets content levels for each hazardous element to identify soil contamination and remediation needs. It distinguishes the so-called ´threshold value´ and ´guideline value´. If the guideline value is exceeded, the area has a contamination level that presents ecological or health risks.

**Table 3:** Total content (T) of heavy metals in the soil depth of 0–10 and 20–30 cm in contaminated, alluvial and reference sites with basic statistical characteristics compared to threshold and guideline values (mg/kg).

Depth (cm)	Site	Locality	Cd	Co	Cr	Cu	Mo	Ni	Pb	Zn
0–10	CS	2CS-PEB	**13.18**	**16.90**	32.72	**90.18**	**12.28**	27.83	**868.39**	**1841.51**
		4CS-CEB	**6.96**	**17.89**	33.39	**81.80**	2.03	22.74	**2924.80**	**979.39**
		*x̄*	*10.07*	*17.40*	*33.05*	*85.99*	*7.15*	*25.29*	*1896.59*	*1410.45*
		*SD*	*3.11*	*0.49*	*0.33*	*4.19*	*5.13*	*2.55*	*1028.21*	*431.06*
	AS	1AS-RZ	**14.16**	**15.68**	24.35	**122.32**	3.83	18.02	**2602.79**	**1948.56**
		1AS-GL	**23.95**	7.98	11.98	**143.71**	3.99	11.98	**890.22**	**2155.69**
		3AS-RZ	**12.14**	11.56	19.58	**117.68**	3.08	12.95	**631.60**	**1566.59**
		5AS-RZ	**3.93**	**16.62**	29.40	**35.26**	1.03	17.86	**401.56**	**454.68**
		6AS-RZ	**15.85**	**17.69**	25.97	**252.70**	1.86	15.94	**919.51**	**2032.25**
		6AS-GL	**11.97**	11.97	23.93	**227.36**	0.00	19.94	**1108.90**	**3103.31**
		8AS-RZ	**14.65**	**17.56**	24.97	**156.93**	2.29	17.53	**602.72**	**1920.56**
		8AS-GL	**3.94**	11.81	23.62	**35.43**	0.00	11.81	**153.54**	**307.09**
		9AS-RZ	**19.57**	13.69	20.10	**235.96**	3.46	13.53	**1245.83**	**2044.74**
		10AS-RZ	**11.81**	**17.35**	22.24	**210.75**	2.90	13.86	**783.27**	**1327.99**
		11AS-RZ	**16.39**	**15.40**	18.46	**266.59**	2.35	13.11	**1042.77**	**1810.83**
		12AS-RZ	**16.06**	13.44	19.26	**222.32**	1.44	12.45	**1357.63**	**1669.09**
		13AS-RZ	**14.06**	**18.09**	34.05	**147.01**	2.66	18.46	**977.43**	**1362.35**
		13AS-AL-1	**11.84**	11.84	35.53	**126.33**	0.00	15.79	**1508.09**	**1267.27**
		13AS-AL-2	**11.96**	**15.94**	51.81	**151.45**	0.00	27.90	**1466.72**	**1502.59**
		14AS-AL	**14.73**	12.63	28.79	**95.80**	1.16	15.69	**1512.26**	**1653.78**
		15AS-RZ	**11.87**	11.87	55.38	**106.80**	0.00	27.69	**439.08**	**1079.91**
		15AS-AL-1	**23.71**	**15.80**	43.46	**537.34**	3.95	23.71	**2580.01**	**2370.60**
		15AS-AL-2	**19.67**	13.95	62.11	**170.33**	1.57	34.78	**1104.50**	**2106.45**
		*x̄*	*14.33*	*14.26*	*30.26*	*176.95*	*1.87*	*18.05*	*1122.55*	*1667.60*
		*SD*	*5.12*	*2.66*	*13.32*	*106.78*	*1.41*	*6.14*	*627.45*	*628.24*
		*Min*	*3.93*	*7.98*	*11.98*	*35.26*	*0.00*	*11.81*	*153.54*	*307.09*
		*Max*	*23.95*	*18.09*	*62.11*	*537.34*	*3.99*	*34.78*	*2602.79*	*3103.31*
	RS	7RS-GL	**3.33**	**15.20**	24.92	13.48	1.55	14.70	**94.87**	**170.25**
20–30	AS	1AS-RZ	**14.20**	13.98	21.95	**127.28**	3.85	14.77	**1081.98**	**1744.37**
		3AS-RZ	**13.18**	13.11	21.80	**134.37**	3.77	14.67	**506.67**	**1710.54**
		5AS-RZ	**3.48**	13.69	30.03	**30.42**	0.72	16.44	**377.30**	**354.14**
		6AS-RZ	**6.52**	14.70	20.98	**327.33**	2.45	12.72	**779.92**	**801.32**
		8AS-RZ	**10.86**	**17.72**	21.73	**144.88**	1.92	15.92	**607.55**	**1241.98**
		9AS-RZ	**13.83**	14.53	27.62	**186.04**	1.20	15.16	**953.02**	**1769.38**
		10AS-RZ	**11.28**	**18.86**	23.40	**226.91**	1.95	15.04	**1032.18**	**1570.78**
		11AS-RZ	**14.54**	**15.01**	22.71	**238.26**	2.50	13.45	**995.32**	**1812.79**
		12AS-RZ	**23.27**	14.51	17.71	**308.42**	2.31	13.00	**1262.08**	**2033.20**
		13AS-RZ	**13.61**	**19.13**	25.86	**140.62**	2.31	17.43	**867.61**	**1250.05**
		14AS-AL	**15.78**	**15.20**	27.24	**111.08**	1.65	16.77	**1425.55**	**1857.01**
		15AS-AL-2	**18.03**	13.29	58.91	**173.34**	1.23	33.86	**1104.08**	**2131.51**
		*x̄*	*13.21*	*15.31*	*26.66*	*179.08*	*2.16*	*16.60*	*916.10*	*1523.09*
		*SD*	*4.85*	*2.00*	*10.25*	*80.95*	*0.91*	*5.39*	*294.00*	*501.15*
		*Min*	*3.48*	*13.11*	*17.71*	*30.42*	*0.72*	*12.72*	*377.30*	*354.14*
		*Max*	*23.27*	*19.13*	*58.91*	*327.33*	*3.85*	*33.86*	*1425.55*	*2131.51*
	RS	7RS-GL	**3.94**	11.83	23.67	23.67	0.00	11.83	**110.45**	**177.51**
*Limits*		*1*	***0.4–1.0***	***15–20***	***100–200***	***30–70***		***40–60***	***25–115***	***100–200***
		*2*	***1***	***20***	***100***	***100***		***50***	***60***	***200***
		*3*	***10–20***	***100–250***	***200–300***	***150–200***		***100–150***	***200–750***	***250–400***
		*4*	***1.4***	***40***	***64***	***63***	***5***	***45***	***70***	***250***
		*5*	***22***	***300***	***87***	***91***	***40***	***89***	***600***	***410***

*AS* alluvial site, *CS* contaminated site, *RS* reference site (used to determine background values), *RZ* riparian zone, *AL* arable land, *GL* grassland, *PEB* probable environmental burden, *CEB* confirmed environmental burden. Signs mean: 1—Slovak threshold value of soil contamination in agricultural soil, 2—Finish threshold value of soil contamination, 3—Finish guideline values of soil contamination and remediation needs, 4—Canadian soil quality guidelines for the protection of environmental and human health for agricultural land, 5—Canadian soil quality guidelines for the protection of environmental and human health for industrial land. Normal letter means below all threshold values; bold letter means above at least one threshold value.

For all HMs, except Cr and Ni, we recorded at least one locality exceeding one of the limit values. In the case of Mo, the total content exceeded the limit value only at one CS locality. The total Co content slightly exceeded only the Slovak limit value at CS localities and several AS localities, and was below other national limit values. We recorded 4 HMs (Cd, Cu, Pb, Zn) whose total content in most localities significantly exceeded all limit values, including the Finnish guideline values for soils with decontamination and remediation needs. At AS localities, the situation in total HM content of both observed soil depths was similar. HM contamination was also observed across broader floodplains.

### Contamination and ecological risk assessment for heavy metals in soil

Contamination and ecological risk at the CS and AS localities were confirmed by calculated indices along the entire Štiavnica River floodplain including the downstream part of the Podunajská pahorkatina Hills (Table 4).

**Table 4:** Contamination factor (CF), pollution load index (PLI), potential ecological risk factor (Er), potential ecological risk of the environment (RI) for contaminated and alluvial sites in the soil depth of 0–10 cm.

Site	Locality	CF								PLI	Er				RI
		Cd	Co	Cr	Cu	Mo	Ni	Pb	Zn		Cd	Cu	Pb	Zn	
CS	2CS-PEB	4.0	1.1	1.3	6.7	7.9	1.9	4.8	10.8	3.1	120.0	33.5	24.0	10.8	188.3
	4CS-CEB	2.1	1.2	1.3	6.1	1.3	1.5	5.5	5.8	2.0	63.0	30.5	27.5	5.8	126.8
	*x̄*	*3.0*	*1.1*	*1.3*	*6.4*	*4.6*	*1.7*	*5.2*	*8.3*	*2.6*	*91.5*	*32.0*	*25.8*	*8.3*	*157.6*
AS	1AS-RZ	4.3	1.0	1.0	9.1	2.5	1.2	27.4	11.4	3.1	129.0	45.5	137.0	11.4	322.9
	1AS-GL	7.2	0.5	0.5	10.7	2.6	0.8	9.4	12.7	2.4	216.0	53.5	47.0	12.7	329.2
	3AS-RZ	3.6	0.8	0.8	8.7	2.0	0.9	6.7	9.2	2.2	108.0	43.5	33.5	9.2	194.2
	5AS-RZ	1.2	1.1	1.2	2.6	0.7	1.2	4.2	2.7	1.4	36.0	13.0	21.0	2.7	72.7
	6AS-RZ	4.8	1.2	1.0	18.7	1.2	1.1	9.7	11.9	2.9	144.0	93.5	48.5	11.9	297.9
	6AS-GL	3.6	0.8	1.0	16.9	0.0	1.4	11.7	18.2	0.8	108.0	84.5	58.5	18.2	269.2
	8AS-RZ	4.4	1.2	1.0	11.6	1.5	1.2	6.4	11.3	2.7	132.0	58.0	32.0	11.3	233.3
	8AS-GL	1.2	0.8	0.9	2.6	0.0	0.8	1.6	1.8	0.4	36.0	13.0	8.0	1.8	58.8
	9AS-RZ	5.9	0.9	0.8	17.5	2.2	0.9	13.1	12.0	2.9	177.0	87.5	65.5	1.2	342.0
	10AS-RZ	3.5	1.1	0.9	15.6	1.9	0.9	8.3	7.8	2.6	105.0	78.0	41.5	7.8	232.3
	11AS-RZ	4.9	1.0	0.7	19.8	1.5	0.9	11.0	10.6	2.7	147.0	99.0	55.0	10.6	311.6
	12AS-RZ	4.8	0.9	0.8	16.5	0.9	0.8	14.3	9.8	2.5	144.0	82.5	71.5	9.8	307.8
	13AS-RZ	4.2	1.2	1.4	10.9	1.7	1.3	10.3	8.0	2.7	126.0	54.5	51.5	8.0	240.0
	13AS-AL-1	3.6	0.8	1.4	9.4	0.0	1.1	15.9	7.4	0.7	108.0	47.0	79.5	7.4	241.9
	13AS-AL-2	3.6	1.0	2.1	11.2	0.0	1.9	15.5	8.8	0.8	108.0	56.0	77.5	8.8	250.3
	14AS_AL	4.4	0.8	1.2	7.1	0.7	1.1	15.9	9.7	2.3	132.0	35.5	79.5	9.7	256.7
	15AS-RZ	3.6	0.8	2.2	7.9	0.0	1.9	4.6	6.3	0.7	108.0	39.5	23.0	6.3	176.8
	15AS-AL-1	7.1	1.0	1.7	39.8	2.5	1.6	27.2	13.9	4.1	213.0	199.0	136.0	13.9	561.9
	15AS-AL-2	5.9	0.9	2.5	12.6	1.0	2.4	11.6	12.4	3.0	177.0	63.0	58.0	12.4	310.4
	*x̄*	*4.3*	*0.9*	*1.2*	*13.1*	*1.2*	*1.2*	*11.8*	*9.8*	*2.2*	*129.2*	*65.6*	*59.2*	*9.8*	*263.7*
	*SD*	*1.5*	*0.18*	*0.5*	*7.9*	*0.9*	*0.4*	*6.6*	*3.7*	*1.0*	*45.9*	*39.6*	*33.0*	*3.7*	*103.7*
	*Min*	*1.2*	*0.5*	*0.5*	*2.6*	*0.0*	*0.8*	*1.6*	*1.8*	*0.4*	*36.0*	*13.0*	*8.0*	*1.8*	*58.8*
	*Max*	*7.2*	*1.2*	*2.5*	*39.8*	*2.6*	*2.4*	*27.4*	*18.2*	*4.1*	*216.0*	*199.0*	*137.0*	*18.2*	*561.9*

*AS* alluvial site, *CS* contaminated site, *RZ* riparian zone, *AL* arable land, *GL* grassland, *PEB* probable environmental burden, *CEB* confirmed environmental burden. The CF < 1 means low contamination, 1–3 is moderate contamination, 3–6 is considerable contamination and CF > 6 means very high contamination. The PLI > 1 indicates pollution. The Er < 40 means low risk, 40–80 is moderate risk, 80–160 is considerable risk, 160–320 is high risk, and > 320 is very high risk. The RI < 150 means low risk, 150–300 is moderate risk, 300–600 is considerable risk, and > 600 is very high risk.

In contaminated sites, the average CF showed a very high contamination by Zn and Cu; considerable contamination by Cd, Mo, Pb; and moderate contamination by Co, Cr, and Ni. At alluvial sites, the average CF showed very high contamination by Cu, Pb, and Zn; considerable contamination by Cd; moderate contamination by Cr, Mo, and Ni; and low contamination by Co. Very high contamination (CF > 6) was calculated for Pb, Zn, and Cu at most AS localities. Overall, the highest CF was calculated in the case of Cu (39.8 in the 15AS-AL locality), Pb (27.4 in the 1AS-RZ locality), Zn (18.2 in the 6AS-GL locality), Cd (7.2 in the 1AS-GL locality). Contamination by HMs was, in many AS localities, similar or even higher than in CS localities. Metal PLI values confirmed that the CS and AS sites were contaminated except for five AS localities, two of them are used as grasslands, two as arable land, and one is located in the riparian zone. The average Er showed a considerable risk of Cd at the AS and CS localities, and moderate risk of Cu and Pb at the AS localities. The RI values indicated a considerable ecological and environmental risk at 7 study sites located mainly in riparian forests, moderate risk at 11 study sites and only 2 study sites located in the riparian zone were at low risk.

### Heavy metals fractionation

The sequential extraction procedure enables us to measure broader forms or phases.

**Figure 2:**
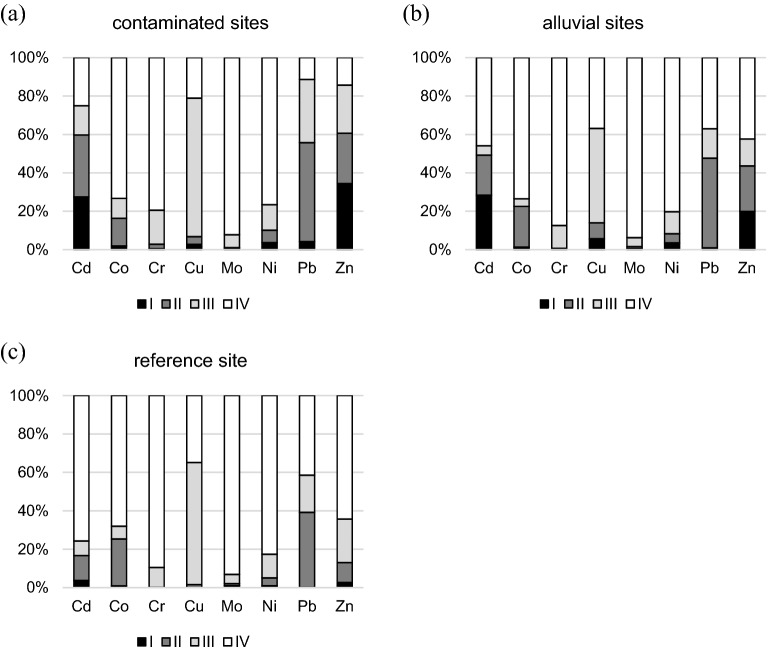
Distribution of potential toxic elements in 4 fractions in the soil depth of 0–10 cm. *I* exchangeable and weak acid soluble fraction, *II* reducible fraction, *III* oxidisable fraction, *IV* residual fraction.

HMs were distributed in all four fractions (exchangeable, reducible, oxidisable, and residual) between the CS, AS, and RS localities (Fig. 2a–c).

The content of HMs in the residual fraction was dominated at the RS localities (in 7 of 8 HMs). The study of the partitioning of HMs in the different fractions at the CS and AS localities revealed that Cd, Cu, Pb, and Zn were mainly associated with the first three fractions. The first exchangeable fraction was the highest in the case of Cd and Zn. Cu was mainly associated with the oxidisable fraction bound to organic matter and sulphides. The distribution of HMs in the different fractions, mainly in exchangeable ones, suggested that the mobility and bioavailability of the metals probably declined in the order of Cd, Zn, Cu, and Pb at AS localities, and Zn, Cd, Pb, and Cu at CS localities.

The four most serious fractions of pollutants (Cd, Cu, Pb, Zn) in the Štiavnica River floodplain indicate their different distribution patterns. At AS localities, on average, the content of Cd represented 46.0% in residual, 28.4% in exchangeable, 20.9% in reducible, and 4.9% in oxidisable fraction. At CS localities, the highest percentages of Cd were associated with the reducible (32.2%), exchangeable (27.4%), residual (25.0%) and oxidisable (15.2%) fraction.

At AS localities, on average, the Cu content accounted for 49.2% in oxidisable, 36.8% in residual, 8.3 in reducible, and 5.7% in exchangeable fraction. At CS localities, the share of different fractions was similar, but the Cu share bond on organic matter and sulphides was higher (72.1%).

At AS localities, on average, the content of Pb represented 46.7% in reducible, 37.0% in residual, 15.3 in oxidisable, and 1.0% in exchangeable fraction. At CS localities, the share of different fractions was different, 51.5% in reducible, 32.9% in oxidisable, 4.2% in exchangeable, and 11.3% in residual fraction.

At AS localities, on average, the content of Zn represented 42.3% in residual, 23.7% in reducible, 19.9% in exchangeable, and 14.1% in oxidisable fraction. At CS localities, the share of the different fractions was different, 34.4% in exchangeable, 26.2 in reducible, 25.1% in oxidisable, and 14.3% in residual fraction.

### Relationships between HM fractions, soil properties, ecosystem types, and soil depth

The soil chemical and physical properties play an important role in the binding and migration of HMs in the soil. The correlation coefficient matrix for the HMs chemical fractions with the soil chemical and physical properties are shown in Tables 5 and 6.

**Table 5:** Pearson’s correlation coefficient matrix of HMs in various fractions and soil chemical properties.

		pH/KCl	Eh	Fe_o_	Mn_o_	SOC	HA/FA
Cd	I	N.C	N.C	N.C	N.C	N.C	N.C
	II	0.472**	N.C	0.547**	0.566**	N.C	N.C
	III	0.464**	N.C	0.533**	0.410*	N.C	N.C
	IV	N.C	N.C	− 0.468**	N.C	N.C	N.C
	T	N.C	N.C	N.C	N.C	N.C	N.C
Co	I	N.C	N.C	N.C	− 0.362*	N.C	N.C
	II	N.C	N.C	0.363*	N.C	N.C	N.C
	III	0.373*	N.C	0.784**	0.558**	N.C	N.C
	IV	N.C	N.C	N.C	N.C	N.C	N.C
	T	N.C	N.C	0.423*	N.C	N.C	N.C
Cr	I	0.537**	N.C	N.C	N.C	N.C	N.C
	II	0.476**	N.C	0.413*	N.C	N.C	N.C
	III	0.471**	N.C	N.C	N.C	N.C	0.396*
	IV	N.C	N.C	− 0.397*	N.C	N.C	N.C
	T	N.C	N.C	− 0.336*	N.C	N.C	N.C
Cu	I	− 0.344*	N.C	N.C	N.C	− 0.486**	− 0.430**
	II	N.C	N.C	0.445**	N.C	− 0.557**	− 0.354*
	III	N.C	N.C	0.503**	N.C	N.C	N.C
	IV	N.C	N.C	N.C	N.C	N.C	N.C
	T	N.C	N.C	N.C	N.C	N.C	N.C
Mo	I	0.336*	N.C	0.464**	0.368*	N.C	N.C
	II	0.461**	N.C	0.573**	0.794**	N.C	0.457**
	III	0.485**	N.C	N.C	0.457**	N.C	N.C
	IV	0.499**	N.C	0.494**	0.537**	N.C	N.C
	T	0.508**	N.C	0.493**	0.548**	N.C	N.C
Ni	I	N.C	N.C	N.C	N.C	N.C	N.C
	II	0.523**	N.C	0.386*	0.418*	N.C	0.353*
	III	0.526**	N.C	N.C	N.C	N.C	0.360*
	IV	N.C	N.C	N.C	N.C	N.C	N.C
	T	0.411*	N.C	N.C	N.C	N.C	N.C
Pb	I	N.C	N.C	N.C	N.C	N.C	N.C
	II	N.C	N.C	N.C	N.C	N.C	N.C
	III	0.538**	N.C	N.C	N.C	N.C	0.414*
	IV	N.C	N.C	N.C	N.C	N.C	N.C
	T	0.368*	N.C	N.C	N.C	N.C	0.373*
Zn	I	0.353*	N.C	0.431**	0.346*	N.C	N.C
	II	0.460**	N.C	N.C	N.C	N.C	N.C
	III	0.487**	N.C	N.C	N.C	N.C	0.340*
	IV	N.C	N.C	N.C	N.C	N.C	N.C
	T	0.342*	N.C	N.C	N.C	N.C	N.C

*I* exchangeable fraction, *II* reducible fraction, *III* oxidisable fraction, *IV* residual fraction, *T* total content; *r values shown in bold are significant at p < 0.05; **r values shown in bold are significant at p < 0.01, *N.C* no correlation.

**Table 6:** Pearson´s correlation coefficient matrix of HMs in various fractions and soil physical properties.

		Content of			PD	BD	MCWC	FWC	Pt	Pc	Pn	Ps	MAC
		Clay	Silt	Sand									
Cd	I	N.C	N.C	N.C	N.C	N.C	N.C	N.C	N.C	N.C	N.C	N.C	N.C
	II	N.C	N.C	N.C	N.C	N.C	N.C	N.C	N.C	N.C	− 0.466*	N.C	− 0.431*
	III	N.C	− 0.424*	N.C	N.C	N.C	N.C	N.C	N.C	N.C	N.C	N.C	N.C
	IV	N.C	N.C	N.C	N.C	− 0.493**	N.C	N.C	0.508**	N.C	0.489**	N.C	0.493**
	T	N.C	N.C	N.C	N.C	N.C	N.C	N.C	N.C	N.C	N.C	N.C	N.C
Co	I	0.509**	N.C	− 0.340*	N.C	N.C	N.C	N.C	N.C	N.C	N.C	N.C	N.C
	II	N.C	N.C	N.C	N.C	N.C	N.C	N.C	N.C	N.C	− 0.394*	N.C	N.C
	III	N.C	N.C	N.C	N.C	N.C	N.C	N.C	N.C	N.C	− 0.421*	N.C	− 0.375*
	IV	N.C	N.C	N.C	N.C	N.C	− 0.403*	− 0.484**	N.C	− 0.484**	0.495**	N.C	0.494**
	T	N.C	− 0.363*	0.373*	N.C	N.C	N.C	− 0.430*	N.C	− 0.430*	N.C	0.467*	N.C
Cr	I	N.C	N.C	N.C	N.C	N.C	N.C	N.C	N.C	N.C	N.C	N.C	N.C
	II	N.C	N.C	N.C	N.C	N.C	N.C	N.C	N.C	N.C	N.C	N.C	N.C
	III	0.657**	N.C	-0.445**	N.C	N.C	N.C	N.C	N.C	N.C	N.C	N.C	N.C
	IV	0.579**	0.480**	-0.611**	N.C	N.C	− 0.473**	N.C	N.C	N.C	N.C	− 0.550**	N.C
	T	0.626**	0.464**	-0.619**	N.C	N.C	− 0.433*	N.C	N.C	N.C	N.C	− 0.540**	N.C
Cu	I	N.C	− 0.410*	0.421*	N.C	N.C	N.C	− 0.435*	0.381*	− 0.435*	0.483**	0.384*	0.519**
	II	N.C	− 0.533**	0.420*	N.C	N.C	N.C	N.C	N.C	N.C	N.C	0.527**	N.C
	III	N.C	− 0.552**	0.496**	N.C	N.C	N.C	N.C	N.C	N.C	N.C	0.428*	N.C
	IV	N.C	N.C	N.C	N.C	N.C	N.C	N.C	0.381*	N.C	0.544**	N.C	0.542**
	T	N.C	N.C	N.C	N.C	N.C	N.C	N.C	0.389*	N.C	0.404*	N.C	0.438*
Mo	I	N.C	− 0.356*	N.C	N.C	N.C	N.C	N.C	N.C	N.C	N.C	N.C	N.C
	II	N.C	N.C	N.C	N.C	N.C	N.C	N.C	N.C	N.C	N.C	N.C	N.C
	III	N.C	N.C	N.C	N.C	N.C	N.C	N.C	N.C	N.C	N.C	N.C	N.C
	IV	N.C	− 0.353*	0.372*	N.C	N.C	N.C	N.C	N.C	N.C	N.C	N.C	N.C
	T	N.C	− 0.345*	0.361*	N.C	N.C	N.C	N.C	N.C	N.C	N.C	N.C	N.C
Ni	I	N.C	N.C	N.C	N.C	N.C	N.C	N.C	N.C	N.C	N.C	N.C	N.C
	II	0.374*	N.C	N.C	N.C	N.C	N.C	N.C	− 0.407*	N.C	− 0.486**	N.C	− 0.467*
	III	0.693**	N.C	-0.509**	N.C	N.C	N.C	N.C	N.C	N.C	N.C	N.C	N.C
	IV	0.432**	0.387*	-0.478**	N.C	N.C	− 0.486**	N.C	N.C	N.C	N.C	− 0.456*	N.C
	T	0.573**	0.353*	-0.511**	N.C	N.C	− 0.369*	N.C	N.C	N.C	N.C	− 0.447*	N.C
Pb	I	N.C	N.C	N.C	N.C	N.C	− 0.397*	N.C	N.C	N.C	N.C	N.C	N.C
	II	N.C	N.C	N.C	N.C	N.C	− 0.456**	− 0.413*	N.C	− 0.413*	N.C	N.C	N.C
	III	N.C	N.C	N.C	N.C	N.C	N.C	N.C	N.C	N.C	N.C	N.C	N.C
	IV	N.C	N.C	N.C	N.C	N.C	N.C	N.C	N.C	N.C	N.C	N.C	N.C
	T	N.C	N.C	N.C	N.C	N.C	N.C	N.C	N.C	N.C	N.C	N.C	N.C
Zn	I	− 0.424*	− 0.493**	0.555**	N.C	N.C	N.C	N.C	N.C	N.C	N.C	N.C	N.C
	II	N.C	N.C	N.C	N.C	N.C	N.C	N.C	N.C	N.C	N.C	N.C	N.C
	III	N.C	N.C	N.C	N.C	N.C	N.C	N.C	N.C	N.C	N.C	N.C	N.C
	IV	N.C	N.C	N.C	N.C	N.C	N.C	N.C	N.C	N.C	N.C	N.C	N.C
	T	N.C	N.C	N.C	N.C	N.C	N.C	N.C	N.C	N.C	N.C	N.C	N.C

*I* exchangeable fraction, *II* reducible fraction, *III* oxidisable fraction, *IV* residual fraction, *T* total content, *r values shown in bold are significant at p < 0.05, **r values shown in bold are significant at p < 0.01, *N.C* no correlation.

Soil properties were selectively correlated with the HM fractions. The most numerous significant correlations with chemical properties occurred in the case of Mo (15), while the least occurred in the case of Pb (4). The most numerous significant correlations with physical properties occurred in the case of Cu (20) while the least occurred in the case of Zn (3).

The selected HM fractions of all HMs were significantly positively correlated with pH except for a significant negative correlation with the I Cu fraction. No statistically significant correlations were found between all HM fractions and Eh. The most numerous, mainly positive, correlations were found with the oxalate-extractable Fe and Mn in the case of Mo followed by Cd, Co, and Cr, while they did not occur in the case of Pb. Correlations with SOC occurred only with Cu (I and II fractions). Significantly positive correlations occurred between HA/FA in the case of selected fractions of Cr, Mo, Ni, Pb, Zn and significantly negative only in the case of Cu (I and II fractions) (Table 5).

The most numerous correlations were found with the different particle size fractions in the case of Ni followed by Cr, Cu, Mo, and Co, while they did not occur in the case of Pb. While the selected HM fractions were mostly positively and significantly correlated with the content of clay particles, a reversed situation occurred in the case of the content of sand particles. No statistically significant correlations were found between all HM fractions and PD and only one negative correlation was found between BD and the IV Cd fraction. The water capacity parameters (MCWC and FWC), only negatively correlated with the selected HM fractions of Co, Cr, Cu, Ni, and Pb. The Cd, Mo, and Zn fractions were not correlated with MCWC and FWC. The most numerous correlations were found with the different porosity categories and MAC in the case of selected fractions of Cu followed by Co, Ni, and Cd (Table 6).

**Figure 3:**
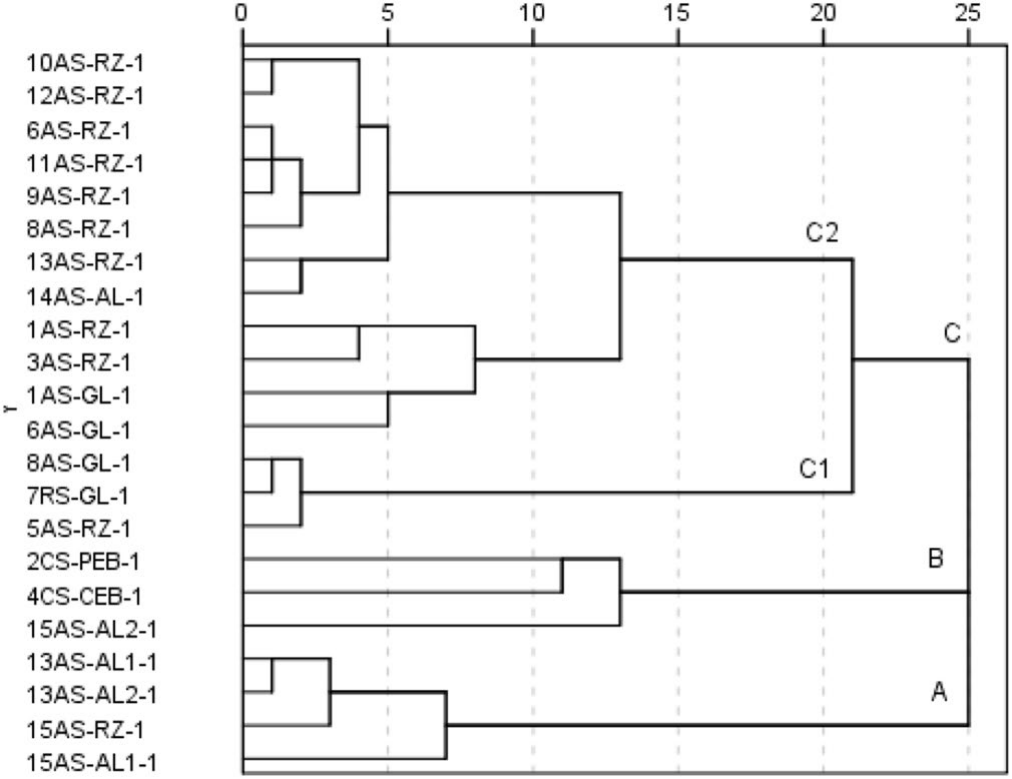
Dendrogram for hierarchical clustering of sites for the surface soil layer (Ward’s method).

In Fig. 3, the hierarchical dendrogram of the linkage between the localities is shown for the surface soil layer (depth 0–10 cm). The variables taken for this analysis are the same as the Pearson´s correlation analysis.

The HCA has highlighted three large clusters, A, B, and C. Cluster A includes sites located in the lower part of the river located in the Podunajská pahorkatina Hills in the broader floodplains predominantly used as arable land. Cluster B includes two contaminated sites located in the Štiavnické vrchy Mountains and one site located in the lower part of the river situated in the Podunajská pahorkatina Hills in the broader floodplains used as arable land. The other localities are grouped in cluster C. Two clusters of lower hierarchical order can be recognised in C (C1 and C2). Cluster C1 includes three sites located in the middle part of the river located in the Štiavnické vrchy Mountains, of which one site is located within the riparian zone and two sites are used as grasslands. Cluster C2 includes predominantly sites located in the upper and middle part of the river located in the Štiavnické vrchy Mountains within the riparian zones or used as grasslands.

The ANOVA test was conducted to determine whether the differences in the content of the different HM fractions in the soils could be significant between the three ecosystem types (RZ, PG, AL) and the soil depths (0–10, 20–30 cm).

Based on the ANOVA results, significant differences emerged between the different ecosystem types for Cd, Cr, and Pb in fractions I and II, for Cr, Mo, Ni, and Pb of fraction IV, and for Co, Cr, Mo, Ni, and Pb of the total content. Within fraction III, the ecosystem types had an effect on all the observed HMs. The most significant differences between the different ecosystem types and the HM fractions were found in the case of Cr, followed by Pb, Cd, Mo, Ni Co, Cu, and Zn (Table 7). The effect of soil depth on HM fractions was revealed only in the case of Cr(I) and Co(III).

**Table 7:** Effect of ecosystem type and soil depth on heavy metal fractions.

		Ecosystem type		Soil depth	
		*F*-value	*P*-value	*F*-value	*P*-value
I	Cd	5.313	**0.005**	0.068	0.795
	Co	1.611	0.209	0.058	0.811
	Cr	16.747	**0.000**	9.655	**0.004**
	Cu	1.243	0.313	0.010	0.921
	Mo	2.765	0.060	0.512	0.480
	Ni	0.210	0.889	0.171	0.683
	Pb	7.054	**0.001**	0.124	0.728
	Zn	2.818	0.057	0.435	0.515
II	Cd	3.314	**0.034**	1.615	0.214
	Co	1.927	0.148	0.032	0.859
	Cr	16.518	**0.000**	0.800	0.379
	Cu	1.809	0.168	0.133	0.718
	Mo	1.607	0.210	1.010	0.324
	Ni	1.931	0.147	0.341	0.564
	Pb	12.993	**0.000**	0.689	0.414
	Zn	1.605	0.211	0.050	0.825
III	Cd	5.131	**0.006**	0.205	0.655
	Co	10.588	**0.000**	8.241	**0.008**
	Cr	5.457	**0.004**	0.463	0.502
	Cu	3.525	**0.028**	0.008	0.928
	Mo	5.728	**0.003**	0.001	0.975
	Ni	3.900	**0.019**	0.061	0.807
	Pb	10.466	**0.000**	0.124	0.728
	Zn	5.365	**0.005**	0.000	0.984
IV	Cd	1.002	0.407	5.065	0.032
	Co	0.788	0.511	0.217	0.645
	Cr	5.564	**0.004**	0.064	0.802
	Cu	0.531	0.665	2.511	0.124
	Mo	5.790	**0.003**	0.271	0.607
	Ni	4.084	**0.016**	0.348	0.560
	Pb	0.364	0.780	1.894	0.180
	Zn	1.745	0.181	3.808	0.061
T	Cd	2.311	0.098	0.915	0.347
	Co	3.975	**0.018**	0.020	0.888
	Cr	6.265	**0.002**	0.021	0.885
	Cu	1.621	0.207	0.876	0.357
	Mo	5.912	**0.003**	0.214	0.647
	Ni	6.855	**0.001**	0.137	0.714
	Pb	4.175	**0.015**	1.435	0.241
	Zn	1.891	0.154	1.490	0.232

The P-values highlighted in bold are highly statistically significant (P < 0.001) and statistically significant (P < 0.05).

## Discussion

The high content of HMs in different fractions in floodplain soils along the entire Štiavnica River watercourse proved to be a consequence of past mining activities. Many regions in Europe suffer from soil contamination caused by past mining activities^24^. On the other hand, in many developing countries, soil HM contamination has also become a severe problem due to rapid industrialization over the past several decades^25^.

Based on the findings, Cd, Cu, Pb and Zn are the most serious pollutants of the Štiavnica River floodplain. These metals are listed as priority control pollutants by the US Environmental Protection Agency due to their potential toxic, persistent, and irreversible characteristics. Therefore, their excessive accumulation in soils is of particular interest^26^. The total content of Cd, Cu, Pb, and Zn in most localities significantly exceeded all limit values, including the Finnish guideline values. The Finnish standard values are considered a good approximation of the mean values of the different national systems in Europe and were used by Tóth et al.^27^ for the assessment of agricultural soils contamination in the EU. Contamination factors confirmed that the contamination by HMs at many AS localities was similar or even higher than at CS localities. The mine tailings contain residues characterised by a high content of HMs. The tailings were usually deposited untreated and left without improper management, being so unstable and prone to wind and water erosion^28,29^. HMs were generated during active mining in the eighteenth and nineteenth centuries and later released and transported by watercourse, and subsequently dispersed during floods along the entire Štiavnica River floodplain. This fact was revealed by HCA showing grouping localities on the basis of their similarities. Many of the study sites were under considerable ecological risk to the environment. Nevertheless, some of the localities located in the downstream part of the Podunajská pahorkatina Hills are still used intensively in agriculture.

The natural characteristics of the basin and the water course, including steepness and flow rate, have a significant impact on contamination, and they spread to the more distant surroundings. In the Štiavnické vrchy Mountains, the Štiavnica River flow is steep, narrow, and relatively shallow. In the downstream part of the basin in the Podunajská pahorkatina Hills, the Štiavnica River flow is milder, wider, and deeper. This situation supports heavy metal accumulation downstream, mainly in the part of the floodplain used for agriculture. In addition to the influence of anthropogenic activities, the higher content of clay particles could be the important environmental factor causing the absorption and adsorption of HM by soil. HM accumulated to toxic levels in agricultural land can potentially pose an environmental risk to other ecosystems and food production. Although the Štiavnica River is rather small and reflects the ecological conditions of its local environment and has an impact on the surrounding ecosystems and larger watercourses. Small rivers can have a big effect on larger water bodies and their surroundings^30,31^.

HM toxicity is related not only to the total content in the soil, but also to the distribution of its fractions. It is accepted that a large and relatively stable portion of HMs is present in the crystal lattice of minerals and the residual fraction. On the contrary, it is the mobile exchangeable fraction that can be used to evaluate the extent of environmental bioavailability. Among the HM fractions, the exchangeable fraction determines the most the actual environmental risk, and the reducible and oxidizable ones are the potentially bioavailable fractions. FES in floodplains is directly dependent on the capacity of soil structures to immobilize these mobile and/or potentially mobile and bioavailable fractions. Therefore, it is important to control the bioavailable fractions also for soil remediation^32^. In terms of bioavailability, various species of metals are biologically more available than others. In our study, the content of Cd, Pb, and Zn in all, and Cu in most localities exceeded the Slovak threshold values for mobile forms of HMs in agricultural soils. This suggests that these elements can be readily available to plants and soil organisms^33^. Most HMs are toxic to humans, even at low contents.

The content of HMs in the residual fraction dominated at the RS locality. HMs bound in the residual fraction are naturally present in the parent rock. HMs of anthropogenic input tend to reside commonly in the first three fractions^34^ and this is what we observed at the CS and AS localities in the case of Cd, Cu, Pb, and Zn. In the case of Cd, the results indicated the relative importance of Fe–Mn oxide bound, easily soluble, and exchangeable fractions for Cd^35^. The usual distribution of Cd in contaminated soils is exchangeable > reducible > oxidisable > residual fraction^36,37^ what, in our study, was more similar to the HM fractionation at the CS localities. In the case of Cu, we observed the great importance of organic matter bound, similar to what other authors have observed^26,38^. In the case of Pb, we found the relative importance of Fe and Mn oxide bound, in agreement with the findings of many authors^39–41^.

The correlation analysis allowed us to indicate the key factors that determine the binding of HMs in the soils and their mutual interactions. Many studies have dealt with the correlation relationships between HMs and physicochemical properties^42–44^. However, the results obtained are not unequivocal^45^ but can still serve as a valuable source of information for the management of contaminated sites.

Although soil pH is considered to affect the chemical composition of soil solution and also the bioavailability of heavy metals^46,47^, we found also no correlations between the I HM fraction and the pH in the case of Cd, Co, Ni and Pb. Furthermore, in our study, no correlations with Eh were found, although heavy metal behaviour is strongly dependent on redox gradients^46^. Fe and Mn can strongly adsorb divalent HM cations, and their positive correlation with HMs is more prevalent in the studies^48^ similar to our study. Negative correlation is less common^49^.

In our study, we confirmed the importance of the content of clay and silt particles as well as SOC as sorbents with great absorptive capacity that support FES. This is consistent with the findings of other authors^50^ who claimed that SOC and clay contents^51^ have substantial influences on the variability of the HMs. The grain size is one of the essential factors influencing HM contents in soil^52^. The I fraction of Cu, Mo, and Zn is negatively correlated with the content of clay or silt particles, and the I fraction of Cu is negatively correlated with SOC. Thus, a higher clay and silt content can immobilize metals, such as Cu, Mo, and Zn, and a higher organic content can immobilize metals such as Cu. This finding confirmed the importance of organic soil amendments used to immobilize HMs in soils by changing the speciation from initially highly bioavailable forms to much less bioavailable fractions associated with organic matter^53^. Organic matter is known to have significant immobilizing effects on HMs including Cu^54,55^. Therefore, the retention of HM induced by organic amendment is, on the whole, mainly attributed to an increase in surface charges^56^ and to FES support.

HM interactions with soil hydro-physical properties are not observed and evaluated so often. But these interactions exist and even poor physical properties such as lower soil porosity, and reduced water holding capacity may indirectly result from HM contamination^57^. Zhang et al.^44^ similarly to our results, consider soil porosity as one of the dominant factors determining the distribution of HMs and their fractions in the micropore-dominated riparian soil.

We recorded significant differences between different ecosystem types for all HMs in different fractions but mainly for Cr and Pb, and no effect of soil depth on HM fractions was revealed, except for Cr (I) and Co (III). This indicates that different types of ecosystem and land-use can influence HMs and their fraction content in soils, as documented by many authors^58,59^.

## Conclusions

Based on our findings, there is a significant degree of HM contamination, particularly Cd, Cu, Pb, and Zn in the floodplain soils around the Štiavnica River. This pollution originated mainly from past mining activities. The entire downstream transport of suspended solids was a source of HMs for distant soils and, furthermore, pollution caused serious contamination to distant agricultural land in Podunajská pahorkatina Hills.

We comprehensively analysed soil properties that affect HM fractionation and bioavailability. The distribution of HMs in the different fractions, mainly in exchangeable ones, suggested that the mobility and bioavailability of the metals probably declined to the order of Cd, Zn, Cu, Ni, and Pb. The dominance or significant portion of HM in the exchangeable fraction indicates a lower or higher FES capacity provided by floodplain soils, also determined by other soil properties. Soil properties were selectively correlated with the HM fractions. Based on the ANOVA results, the effect of different ecosystem types on the HM fractions was revealed. Thus, soil properties and ecosystem types confirmed a special role in the fate of heavy metals.

These results highlight the importance of considering the HM fractionation as well as the soil properties of different ecosystem types, when making strategic decisions to arrange anthropogenic activities and prevent risk to the environment and human health. There is an urgent need to preserve existing floodplains as natural resources and begin to restore them. This study could help managers adopt a more ecosystem-based approach to floodplain management by helping to reduce pollution. Furthermore, we should consider the adverse influences on ecosystems, living organisms, and public health.
